# Comparative Proteomic Analysis of Serum from Pigs Experimentally Infected with *Trichinella spiralis*, *Trichinella britovi,* and *Trichinella pseudospiralis*

**DOI:** 10.3390/pathogens9010055

**Published:** 2020-01-11

**Authors:** Michał Gondek, Agnieszka Herosimczyk, Przemysław Knysz, Małgorzata Ożgo, Adam Lepczyński, Krzysztof Szkucik

**Affiliations:** 1 Department of Food Hygiene of Animal Origin, Faculty of Veterinary Medicine, University of Life Sciences in Lublin, Akademicka 12, 20-950 Lublin, Poland; knysz.przemyslaw@gmail.com (P.K.); krzysztof.szkucik@up.lublin.pl (K.S.); 2 Department of Physiology, Cytobiology and Proteomics, Faculty of Biotechnology and Animal Husbandry, West Pomeranian University of Technology, Klemensa Janickiego 29, 71-270 Szczecin, Poland; agnieszka.herosimczyk@zut.edu.pl (A.H.); malgorzata.ozgo@zut.edu.pl (M.O.); adam.lepczynski@zut.edu.pl (A.L.)

**Keywords:** *Trichinella* spp., pigs, experimental infection, serum proteomics, 2-DE, MALDI-TOF MS

## Abstract

Although the available proteomic studies have made it possible to identify and characterize *Trichinella* stage-specific proteins reacting with infected host-specific antibodies, the vast majority of these studies do not provide any information about changes in the global proteomic serum profile of *Trichinella*-infested individuals. In view of the above, the present study aimed to examine the protein expression profile of serum obtained at 13 and 60 days postinfection (d.p.i.) from three groups of pigs experimentally infected with *Trichinella spiralis*, *Trichinella britovi,* and *Trichinella pseudospiralis* and from uninfected, control pigs by two-dimensional gel electrophoresis (2-DE) followed by matrix-assisted laser desorption/ionization time-of-flight (MALDI-TOF) mass spectrometry. The comparative proteomic analysis of the *T. spiralis* group vs. the control group revealed 5 differently expressed spots at both 13 and 60 d.p.i. Experimental infection with *T. britovi* induced significant expression changes in 3 protein spots at 13 d.p.i. and in 6 protein spots at 60 d.p.i. in comparison with the control group. Paired analyses between the group infected with *T. pseudospiralis* and the uninfected control group revealed 6 differently changed spots at 13 d.p.i. and 2 differently changed spots at 60 d.p.i. Among these 27 spots, 15 were successfully identified. Depending on the *Trichinella* species triggering the infection and the time point of serum collection, they were IgM heavy-chain constant region, antithrombin III-precursor, immunoglobulin gamma-chain, clusterin, homeobox protein Mohawk, apolipoprotein E precursor, serum amyloid P-component precursor, Ig lambda chains, complement C3 isoform X1, and apolipoprotein A-I. Our results demonstrate that various *Trichinella* species and different phases of the invasion produce a distinct, characteristic proteomic pattern in the serum of experimentally infected pigs.

## 1. Introduction

Trichinellosis is a serious foodborne parasitic disease caused by nematodes of the genus *Trichinella*. *Trichinella* parasites complete all stages of development in one host, and two different phases, namely intestinal (enteral) and muscular (parenteral), can be observed during infection. Infection occurs after consumption of raw or undercooked meat or meat products infected with first-stage (L1) muscle larvae (ML) of *Trichinella*. Swallowed larvae are released from muscle tissue upon gastric digestion and subsequently reach the small intestine, where they penetrate intestinal mucosa, undergo four molts, and develop into adult worms (males and females) within two days [1]. After mating, females start to release newborn larvae (NBL) as early as 5–7 days postinfection (d.p.i.). NBL penetrate intestinal walls, enter blood and lymphatic vessels, and migrate via the circulatory system to striated muscles, where they develop into infective ML. It is assumed that on day 17 after infection, muscle larvae become invasive for the next host [2], however, that time strongly depends on the *Trichinella* species and/or strains causing the infection, as well as the species of the host. *Trichinella* spp., settling in the host’s striated muscle cells, induce there several changes on the molecular, structural, and biochemical levels that lead to the formation of a specific structure called “nurse cell-larva complex”. In fact, nurse cell formation includes responses from infected muscle cells through de-differentiation, cell cycle re-entry, and G_2_/M cell cycle suspension, as well as responses from satellite cells through activation, proliferation, and differentiation processes [3]. This extraordinary structure supports the growth and development of ML, protects the parasites against the immune mechanisms of the host, and meets the nutritional and metabolic requirements of the larvae. A mature nurse cell-infective larva complex remains stable, and under such conditions, *Trichinella* is able to survive and maintain its infectivity for many years. Research data presented by Fröscher et al. [4], for instance, showed that live larvae were isolated from a patient with trichinosis even 39 years after infection.

Until today, within the *Trichinella* genus, two main clades named encapsulated and nonencapsulated can be distinguished. This classification is based on the ability of the parasite to induce the formation of a collagen capsule around the nurse cell-larva complex in the host’s striated muscles after infection. The encapsulated clade includes seven species (*T. spiralis-*T1, *T. nativa-*T2, *T. britovi-*T3, *T. murrelli-*T5, *T. nelsoni*-T7, *T. patagoniensis-*T12) and three additional genotypes (*Trichinella* T6, *Trichinella* T8, and *Trichinella* T9), whose taxonomic position has not yet been determined [5,6]. The encapsulated species and genotypes infect only mammals, while species belonging to the nonencapsulated clade can infect mammals and birds (*T. pseudospiralis-*T4) or mammals and reptiles (*T. papue-*T10 and *T. zimbabwensis-*T11) [5]. Among all recognized *Trichinella* species and genotypes, four (i.e., *T. spiralis, T. britovi, T. pseudospiralis, T. nativa)* have been confirmed in different host species (domestic as well as wild animals) in Europe, including Poland.

Although the epidemiological pattern of human trichinellosis is changing, and game or horse meat plays an increasingly important role, it should be emphasized that pork is still considered as the most important source of *Trichinella* infection for humans [7]. Epidemiological data from the years 1986–2009 show that 41% of human trichinellosis cases and outbreaks in Poland were linked with pork meat consumption, while the corresponding figures for Germany and Romania were 83% and as much as 95%, respectively [7]. There is a general agreement that the most frequently detected of the abovementioned *Trichinella* species and genotypes in European pigs (sylvatic and domestic) is *T. spiralis*. According to data provided by 22 European countries to the International *Trichinella* Reference Center over the past 20 years, *T. spiralis* has been confirmed in 82% of *Trichinella-*infected pigs, while *T. britovi*, the second most common species circulating in Europe, has been detected in 18% of infected swine [8]. Furthermore, in recent years, *Trichinella pseudospiralis,* which is believed to be the sole nonencapsulated species circulating in Europe, has been diagnosed in breeding pigs from Slovakia [9], Croatia [10], Bosnia and Herzegovina [11], and Spain [12]. An experimental model has proved that pigs are not a suitable host for *Trichinella nativa*, a fourth species occurring in Europe. As shown in several studies, despite high doses of *Trichinella nativa* used for experimental swine infection (i.e., 10,000 or 20,000 ML per pig), no larvae were observed in pig’s muscles or the infection level was very low (i.e., 0.04 larva/gram muscle tissue or less) [13,14,15].

The methods developed for detection of *Trichinella* spp. infection in pigs can be classified as direct or indirect. The direct tests, i.e., trichinoscopy (tissue compression) and artificial digestion, directly demonstrate encysted *Trichinella* larvae in muscle tissue through microscopy or visualize larvae released from muscles during a digestion procedure. The indirect tests include serological techniques that detect specific anti-*Trichinella* antibodies. Commission Regulation (EU) 2015/1375 indicates the magnetic stirrer method for pooled-sample digestion as the reference technique for official *Trichinella* inspection of meat intended for human consumption from swine, other slaughter animals, and wild boars [16]. Consequently, in most European Union countries, the artificial digestion assay has been implemented to test individual slaughtered pigs for the presence of *Trichinella* spp. as part of a routine post-mortem veterinary examination of swine carcasses.

Although more than 150 years have passed since the discovery of *Trichinella*, many mechanisms accompanying the infection and host–pathogen interactions remain unknown. Furthermore, there are still unresolved problems with the development of intravital diagnostic tools capable of detecting *Trichinella* infection at an early stage in both humans and swine. No effective vaccine for food animals has been developed either. Recently, in veterinary sciences, proteomics has become a powerful post-genomic tool for identifying characteristic protein patterns in various body fluids of healthy animals, as well as those suffering from various viral, bacterial, or parasitic diseases. Some of the aims of proteomic research are the identification of disease-specific protein biomarkers, explanation of the pathogenesis and mechanisms of diseases, assessment of animal welfare, and development of new vaccines. According to literature data, swine serum is one of the most intensively studied matrices by various proteomic techniques. Two-dimensional gel electrophoresis (2-DE) has been used to create reference serum proteomic maps for both healthy adult pigs [17] and growing piglets [18,19]. Furthermore, various proteomic tools such as surface-enhanced laser desorption/ionization time-of-flight mass spectrometry (SELDI TOF-MS); two-dimensional and two-dimensional differential gel electrophoresis (2-DE/2D-DIGE) followed by matrix-assisted laser desorption/ionization time-of-flight mass spectrometry (MALDI-TOF MS); or liquid chromatography coupled with tandem mass spectrometry (LC-MS/MS) have also been applied to evaluate serum proteomic profiles of (1) pigs with acute liver failure [20]; (2) pigs exposed to stress factors, such as insufficient space [21]; (3) pigs suffering from low-dose LPS-induced inflammation [22]; and (4) pigs experimentally or naturally infected with classical swine fever virus [23,24], food-and-mouth disease virus [25] or porcine reproductive and respiratory syndrome virus [26]. However, the vast majority of *Trichinella* and trichinellosis proteomic studies focus on the immunoproteomic approach, in which immunoreactive proteins from various life stages and different parts or organs of the parasite are subjected to in-depth proteomic analysis [27,28,29,30,31,32,33,34]. Furthermore, among various *Trichinella* species, *Trichinella spiralis* is studied most often and treated as a model organism for the entire genus. Consequently, most of the available proteomic studies have made it possible to identify and characterize only *Trichinella spiralis*-stage-specific proteins reacting with infected host-specific IgG antibodies and did not provide any information about changes in the global proteomic serum profile of *Trichinella*-infested individuals. Thus, there is a lack of studies describing changes in the global proteomic profile of swine serum in response to various *Trichinella* species during intestinal and muscular phase of the infection.

In view of the above, the present study aimed to examine the protein expression profile in serum from three groups of pigs experimentally infected with (1) *Trichinella spiralis*, (2) *Trichinella britovi,* and (3) *Trichinella pseudospiralis* and from an uninfected, control group by two-dimensional gel electrophoresis (2-DE) followed by matrix-assisted laser desorption/ionization time-of-flight (MALDI-TOF) mass spectrometry. Our experimental design included three different *Trichinella* species that have been found in naturally infected pigs in Europe and which belong to two different clades: encapsulated and nonencapsulated ones. Moreover, serum samples from two different phases of infection were evaluated.

## 2. Results

### 2.1. Distribution and Intensity of T. spiralis, T. britovi, and T. pseudospiralis Larvae Infection in Muscles of Pigs Experimentally Infected with T. spiralis, T. britovi, and T. pseudospiralis

The results for the distribution and intensity of *Trichinella* larvae infection (lpg) in muscles of pigs experimentally infected with *Trichinella spiralis*, *Trichinella britovi,* and *Trichinella pseudospiralis* are shown in Table 1 as mean values ± SD for particular experimental groups. Muscle larvae were isolated from all pigs experimentally infected with *T. spiralis*, *T. britovi,* and *T. pseudospiralis*. Moreover, the presence of *Trichinella* larvae was confirmed in both analyzed muscles taken from each infected pig. As expected, *Trichinella* larvae were not detected in muscles sampled from control pigs. Despite the lowest dose used for experimental infection, the highest larval burdens were found in muscles taken from the *T. spiralis*-infected group. There were no statistically significant differences in the intensity of *Trichinella* larvae infection between the diaphragm and the tongue (*P* ˃ 0.05) within the experimental groups. However, in the *Trichinella spiralis*-infected group, the highest number of larvae were found in the tongue, whereas in the *Trichinella britovi* and *Trichinella pseudospiralis*-infected groups, the diaphragm was the most heavily parasitized muscle.

### 2.2. Differentially Expressed Serum Proteins in Pigs Experimentally Infected with T. spiralis, T. britovi, and T. pseudospiralis on Day 13 Postinfection

Analysis with the PDQuest software allowed us to detect an average of 180 protein spots per gel, which were further quantified in order to assess the difference in their expression. On this basis, we selected approximately 140 spots with similar locations and stain intensities on each gel analyzed.

**Figure 1:**
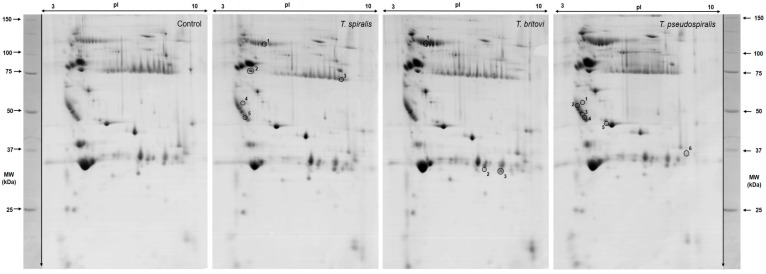
Representative 2-D gel images of differentially expressed protein spots in the serum of pigs experimentally infected with *T. spiralis* (T1), *T. britovi* (T3), and *T. pseudospiralis* (T4) on day 13 postinfection. The differentially expressed spots are numbered and their characterization parameters are given in Table 2 and Figure 3.

**Figure 2:**
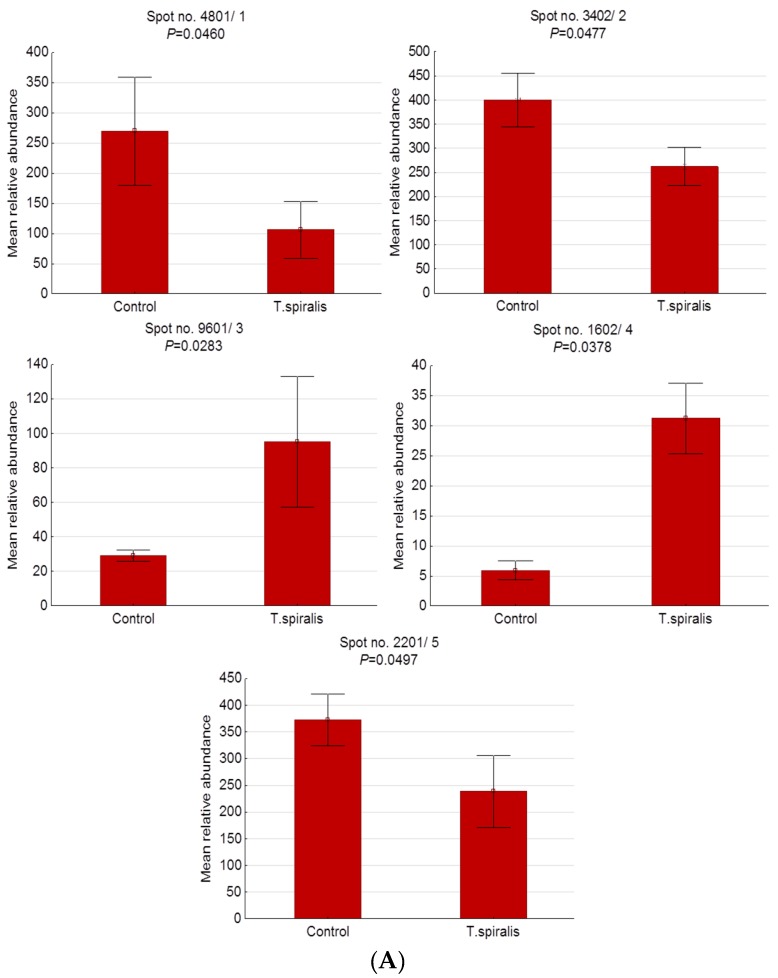

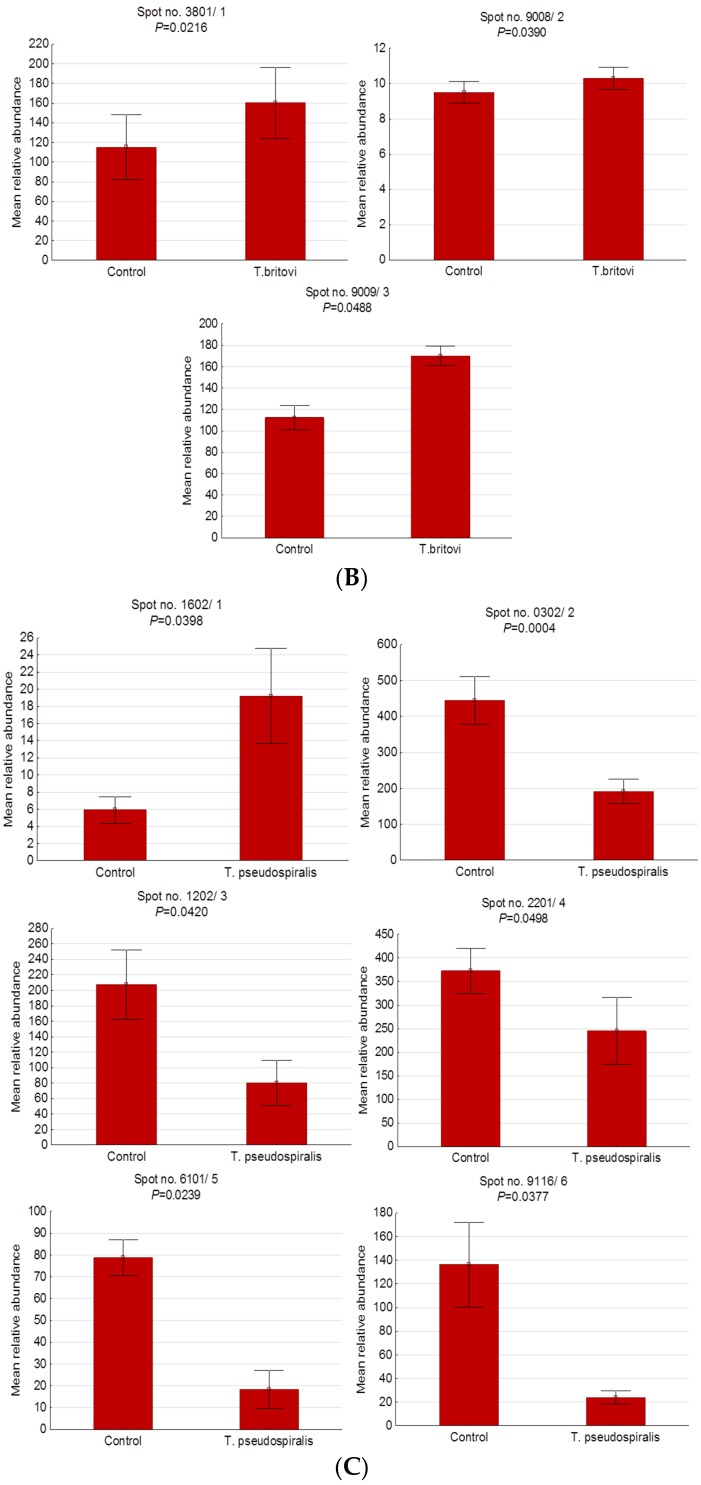
Histograms of protein spots differentially expressed in the serum of uninfected pigs (control) and pigs experimentally infected with *T. spiralis* (**A**), *T. britovi* (**B**), and *T. pseudospiralis* (**C**) on day 13 postinfection. Values are presented as means ± standard deviations.

Figure 1 shows representative 2-D patterns of significantly altered proteins found in pigs experimentally infected with *T. spiralis* (T1), *T. britovi* (T3), and *T. pseudospiralis* (T4). Detailed information concerning the measured 2-DE and MALDI-TOF coordinates of each individual protein spot are provided in Table 2 and Figure 2. All identified serum proteins were categorized according to their cellular localization on the basis of the UniProtKB database (Table 2).

A total of 5 protein spots were differentially expressed in the blood serum of pigs infected with *T. spiralis* at 13 d.p.i. compared to the control group, with 2 up- and 3 downregulated spots. Four of them were successfully identified. Spot no. 4 (not identified) showed the highest expression (5.29-fold increase), whereas spot no. 1, identified as the IgM heavy-chain constant region, was the most underexpressed spot (2.55-fold decrease) in the T1 group.

In the group of pigs infected with *T. britovi*, only 3 spots were statistically altered. All of them were upregulated compared with the control group, with the highest expression (1.51-fold) assigned to the homeobox protein Mohawk (spot no. 3).

A 2-DE analysis revealed 6 differentially expressed protein spots in the T4 group versus the control group. Of these, 1 spot showed increased expression and 5 showed decreased expression. Among successfully identified spots, serum amyloid P-component precursor (spot no. 6) was the most underexpressed spot in this group of pigs (5.68-fold decrease).

### 2.3. Differentially Expressed Serum Proteins of Pigs Experimentally Infected with T. spiralis, T. britovi, and T. pseudospiralis on Day 60 Postinfection

2-D gel analysis allowed us to detect about 130 protein spots per gel, which were further quantified in order to assess the difference in their expression. On this basis, we selected approximately 96 spots that displayed similar locations and stain intensities on each gel analyzed.

**Figure 3:**
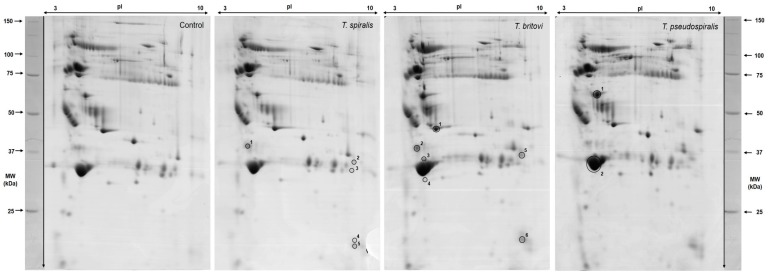
Representative 2-D gel images of differentially expressed protein spots in the serum of pigs experimentally infected with *T. spiralis* (T1), *T. britovi* (T3), and *T. pseudospiralis* (T4) on day 60 postinfection. The differentially expressed spots are numbered and their characterization parameters are given in Table 3 and Figure 4.

**Figure 4:**
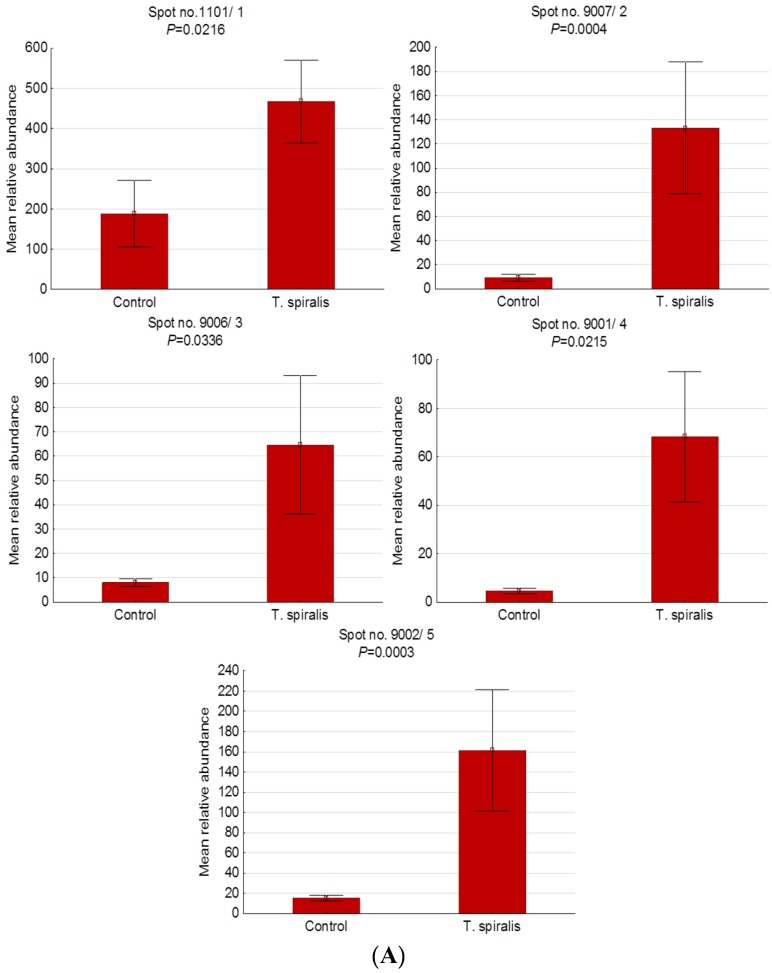

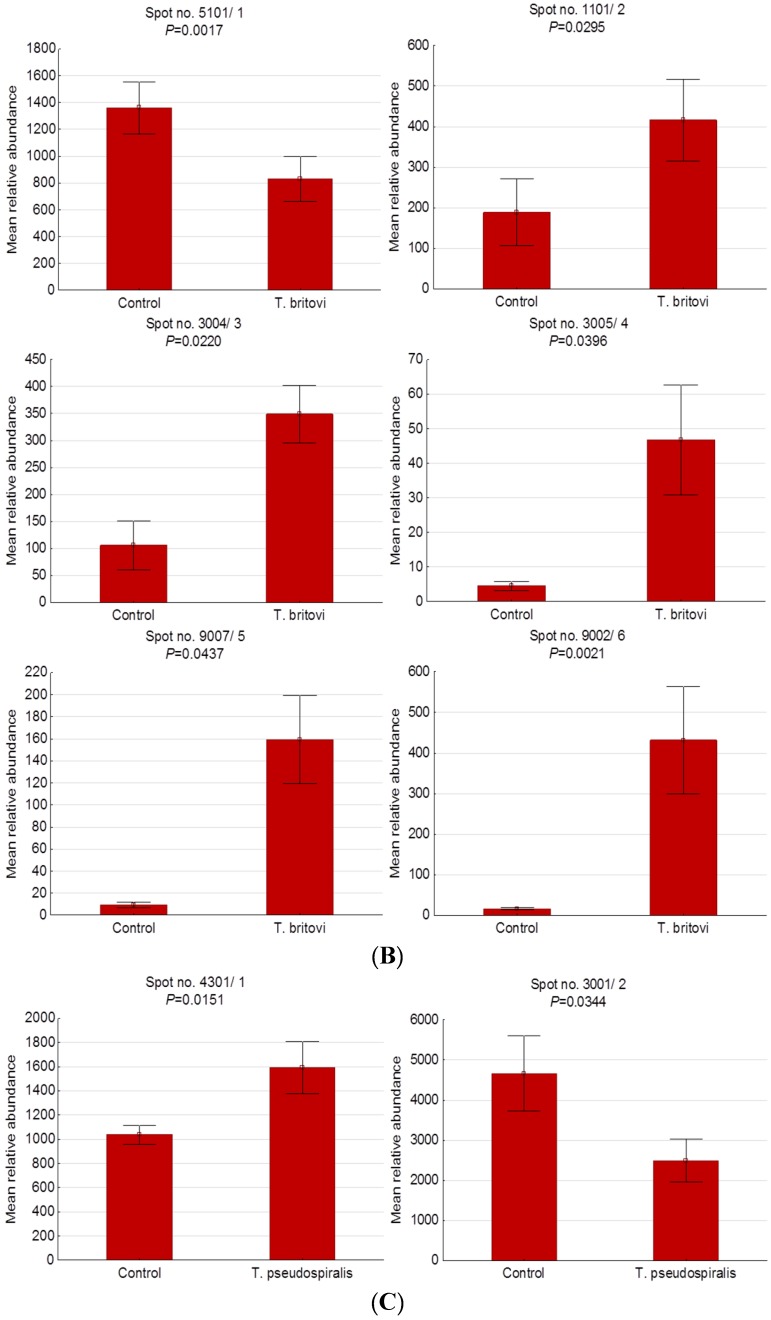
Histograms of protein spots differentially expressed in the serum of uninfected pigs and pigs experimentally infected with *T. spiralis* (**A**), *T. britovi* (**B**), and *T. pseudospiralis* (**C**) on day 60 postinfection. Values are presented as means ± standard deviations.

Figure 3 shows representative 2-D gel images of differentially expressed serum protein spots found in pigs experimentally infected with *T. spiralis* (T1), *T. britovi* (T3), and *T. pseudospiralis* (T4). Lists of proteins and detailed 2-DE and MALDI-TOF data for each protein spot are given in Table 3 and Figure 4. Serum proteins were categorized according to their cellular localization on the basis of the UniProtKB database (Table 3).

Compared with the control group, 5 protein spots were significantly upregulated in the group of pigs infected with *T. spiralis*. Of these, only one was successfully identified (spot no. 1), despite repeated MALDI-TOF analyses. It should be emphasized that the level of expression in this experimental group was highly elevated, with the highest expression assigned to the spot no. 4 (14.87-fold increase).

In pigs infected with *T. britovi,* we have demonstrated changes in the expression of 6 spots, among which only 1 was downregulated, while 5 were shown to be upregulated, in comparison to the control group. In the T3 group, the lowest expression value was observed for apolipoprotein E precursor (spot no. 1), and the highest value was observed for spot no. 6 (not identified). The latter was 28.03 times as high as the corresponding value in the control group at 60 d.p.i.

Only 2 protein spots were significantly altered in the T4 group relative to the C group. Spot no. 1, identified as complement C3 isoform X1, was up-expressed, whereas apolipoprotein A-I (spot no. 2) was down-expressed in response to experimental infection with *T. pseudospiralis*. 

## 3. Discussion

Currently, for food safety purposes and according to the Commission Regulation (EU) 2015/1375 of 10 August 2015, the magnetic stirrer method for pooled-sample digestion is indicated as the reference method applied for the official control of meat intended for human consumption from swine, other species of slaughter animals, and wild boars for *Trichinella*. The sensitivity of the digestion method depends on *Trichinella* ML infection intensity and the amount of muscle sample tested. According to Forbes and Gajadhar [35], for swine muscles, sample size of 1 g allows for the detection of larval loads of ≥3 larvae per gram. In other words, sensitivity equal to 100% was observed when intensity of *Trichinella* ML infection was 3–5 larvae/gram. However, if the infection intensity is 1 larva/gram, the detection rate drops to 73% [36].

Serum contains thousands of proteins produced and released by cells and tissues, and, as a complex biological matrix, it reflects the general health status of the individual. Changes in the serum proteomic pattern were frequently observed during various pathophysiological abnormalities, such as viral, bacterial, or parasitic diseases, in certain food animal species, including pigs [20,21,22,23,24,25,26]. Therefore, farm animal serum proteomics has been developed in order to gain a deeper and more complete insight into the pathomechanism of these health disorders and to find potential biomarkers characteristic of their particular stages.

To the best of our knowledge, there is a lack of studies based on the proteomic approach in which sera from *Trichinella*-infected pigs were assessed to establish parasite-induced changes in their protein profile. Thus, in the present study, we used 2-DE combined with MALDI-TOF MS to determine the characteristic proteomic pattern of serum from pigs experimentally infected with three different *Trichinella* species, namely, *T. spiralis*, *T. pseudospiralis,* and *T. britovi*. To make our study more comprehensive, serum samples from two different phases of the infection were evaluated. Thus, we analyzed serum collected from pigs on day 13 postinfection, as that period largely corresponds to the intestinal phase of the invasion and the preinfective stage of the parasite in the host’s striated muscles. In parallel, we implemented the same procedure for serum samples taken on day 60 postinfection because, according to our previous pilot studies on a swine model and global literature data, that sampling time point corresponds to both the peak of larval density in the host’s muscles and the mature muscle phase with complete invasiveness of the parasite for the next host.

All experimental pigs were successfully infected with *T. spiralis*, *T. pseudospiralis,* and *T. britovi*. To determine the intensity of infection, we chose two different muscles that are considered in pigs as predilection sites of both encapsulated and nonencapsulated clades of *Trichinella*. Our studies confirmed previous reports that *T. spiralis* shows the highest infectivity to domestic pigs, whereas the infectivity of *T. britovi* and *T. pseudospiralis* is rather moderate or low [14,15]. Further, in the experimental groups, there were no statistically significant differences in the intensity of *Trichinella* larvae infection between the diaphragm and the tongue that is confirmed by other authors who identified the diaphragm and the tongue as equally affected muscles with regard to *Trichinella* muscle larvae burden in pigs [37,38,39,40].

### 3.1. Preinfective Stage of T. spiralis, T. britovi, and T. pseudospiralis Larvae in Pig Striated Muscles (13 Day Postinfection)

The proteomic analyses of serum samples obtained on day 13 postinfection from pigs experimentally infected with three different species of *Trichinella* larvae allowed us to visualize, in total, 14 differentially expressed protein spots. At this sampling time point, in the serum of pigs experimentally infected with *T. spiralis*, 3 spots were downregulated and the remaining 2 were upregulated compared to the uninfected control group, and the molecular weight of the significantly altered proteins ranged from 35 kDa to 83.90 kDa. Among the 5 differentially expressed spots in serum samples taken on day 13 postinfection from pigs infected with *T. spiralis*, the MALDI-TOF MS technique allowed us to identify 4: IgM heavy chain, antithrombin III-precursor, immunoglobulin gamma chain, and clusterin. Further, in the serum of pigs experimentally infected with *T. britovi*, which also belongs to the encapsulated clade of *Trichinella*, 3 spots were upregulated, with molecular weights of 23.90 and 84 kDa. We were able to identify 2 of them: heavy chain of immunoglobulin M and homeobox protein Mohawk. In the group of pigs experimentally infected with *T. pseudospiralis*, only 1 protein spot was upregulated and the remaining 5 were significantly downregulated on day 13 postinfection. The molecular weight of the significantly altered proteins in the serum of *T. pseudospiralis*-infected pigs ranged from 25.50 kDa to 40.10 kDa, and they were identified by MALDI TOF MS as clusterin, apolipoprotein E, and serum amyloid P component.

Clusterin, also known as apolipoprotein J (apoJ), is expressed in several tissues and organs, among others, in skeletal muscles, liver, brain, kidney, heart, fat tissue, or testis [41,42,43]. In addition, clusterin has also been identified by 2-dimensional gel electrophoresis in serum samples from healthy pigs of different ages, i.e., 50 days [18] and 4 months [17]. Clusterin can circulate in the blood as a free form or bind to lipoproteins, mainly their HDL fractions and less abundantly to LDL/VLDL [44,45,46]. Although the exact functions of clusterin are not fully understood, a number of recent studies indicate that this protein is involved in several biological processes, including apoptosis [47,48], lipid metabolism [49,50], membrane recycling, complement system regulation [51], or acts as a chaperone protein [52]. Currently, clusterin is attracting scientific attention in the context of neoplasia mechanisms as well. Elevated serum clusterin levels have been observed in the course of many different human disorders, such as diabetes [53], coronary heart disease [53], psoriasis [54], chronic spontaneous urticaria [55], septic shock [56], and several neoplastic diseases, e.g., colorectal cancer [57] or prostate cancer [58]. On the other hand, a decreased serum clusterin level has been proven during osteoarthritis [59], systemic lupus erythematosus [60], sepsis [61], human hepatocellular carcinoma [62], and esophageal squamous cell carcinoma [63]. In the present study, we demonstrated a significantly decreased expression level of clusterin in the serum of pigs experimentally infected with *T. spiralis* and *T. pseudospiralis* on day 13 postinfection as compared to the control swine. Unfortunately, there is no other data on the clusterin level in the serum of *Trichinella*-infected individuals for comparison purposes. However, using cDNA microarray, Wu and colleagues [64] showed that the expression of the clusterin gene in the muscle tissue of mice infected with *Trichinella spiralis* and *Trichinella pseudospiralis* was upregulated. These differences between the results of our proteomic research and the abovementioned transcriptomic one can be explained in several ways. Firstly, these two experimental trials were carried out with samples taken from hosts at different time-points after experimental infection. In our studies, serum samples were collected on day 13 postinfection (the preinfective stage of *Trichinella* L1 larvae in the host’s striated muscles) whereas muscle tissues for gene expression assessment were collected on day 23 after infection (fully mature and invasive *Trichinella* ML larvae in the host’s muscle tissue). Secondly, two different animal models (pigs and mice) were used in these two experiments, and there is sufficient evidence that the gene expression pattern, influenced by damaging factors/infectious agents, strongly depends on the host’s species or cell types [65]. Further, according to data provided by Mido et al. [66], *Trichinella* infection is able to induce the disturbance in lipid metabolism, with a reduction in serum paraoxonase-1 activities and the HDL level and the results of their studies may partially explain apoJ expression changes observed in our experiment as well. Finally, as already mentioned above, clusterin isoform 2 is involved in the regulation of programmed cell death, and its depletion can activate the p53 gene, reduce the expression of Bcl-2 and Bcl-xl family proteins and, as a consequence, make cells more susceptible to the apoptotic action of Bax protein (mitochondrial apoptosis) [67]. It was also shown that during infection with *T. spiralis*, the expression of mitochondrial apoptosis genes, such as BAX, apaf-1, caspase 9, and p53, were increased from day 13 postinfection and reached a peak on day 18 postinfection [68,69]. Based on our studies, it is difficult to explain whether or not clusterin can play an important role in the molecular processes associated with apoptosis during *Trichinella* infection, however, further experiments are needed to confirm or reject this hypothesis.

In the present study, we also found a decreased expression level of antithrombin III precursor (AT III) in the serum of one experimental group only, i.e., in pigs experimentally infected with *T. spiralis*, on day 13 postinfection. Antithrombin III participates in the regulation of blood coagulation, being a major inhibitor of thrombin, factor Xa and, to a lesser extent, factors IXa, XIa, and XIIa [70]. Hence, the deficiency of AT III is a major risk factor for thromboembolic diseases. There are several well-known mechanisms that lead to a decreased antithrombin activity and they can be classified into the following categories: (1) decreased production caused by liver dysfunction or inflammation; (2) increased consumption in the course of disseminated intravascular coagulation (DIC); and (3) increased loss which is observed, e.g., during nephrotic syndrome or gastrointestinal diseases [71]. In addition, according to data provided by Niessen et al. [72], antithrombin III is considered as a negative acute-phase protein. Therefore, we suppose that the downregulation of AT III shown in the present study (on day 13 postinfection) coincides with the acute phase of the invasion, when *Trichinella spiralis* larvae migrate through various organs and tissues and provoke immunological, pathological, and metabolic disturbances with various, often severe, clinical manifestations in humans [1,73,74,75]. For obvious reasons, the available literature provides no information regarding hemodynamic disorders during trichinellosis in pigs. It has been proved, however, that infection with *Trichinella* can induce blood coagulation disorders in humans. In the course of human trichinellosis, the following thrombotic complications were observed: superior sagittal sinus thrombosis was diagnosed in a man infected with *Trichinella spiralis* [76]; ventricular thrombosis occurred 3 weeks after *Trichinella spiralis* infection in a woman [77]; cavernous sinus venous thrombosis complicated by temporary cranial nerve VI palsy was recognized in two patients within two weeks of infection with *Trichinella nativa* larvae [78]. Moreover, disseminated intravascular coagulation in arterioles of various organs has also been described in the course of human trichinellosis [79]. In view of the above, we hypothesize that downregulation of AT III might be involved in hemostatic disorders resulting in thrombosis incidents in the early, acute phase of trichinellosis induced by *T. spiralis.*

Apolipoprotein E (apo E) precursor is another protein, in addition to apo J described above, which is associated with lipid metabolism and whose expression was altered in the present study. A significant downregulation of apo E precursor was found in the serum of *Trichinella pseudospiralis*-infected pigs on day 13 postinfection, as well as in serum samples obtained on day 60 postinfection from pigs infected with *Trichnella britovi*. In mammals, from 60% to 75% of plasma apoE is synthesized in the liver, but other organs, tissues, or cells, such as macrophages, also play a significant role as apo E producers [80,81]. Several various pathomechanisms accompanying *Trichinella* infection should be considered in order to explain apo E decreased expression level found in these studies. As already mentioned above, *Trichinella spiralis* infection evokes lipid metabolism alteration in experimentally infected rats. Further, using rats as a research model, Farid et al. [82] have shown that *Trichinella spiralis* infection is able to induce hepatic inflammation during both intestinal as well as muscular phase of the invasion. Decreases in the level of apolipoproteins (apo E, apo A-I) have also been shown during various liver dysfunctions [83,84]. Hence, we suppose that apo E downregulation might be associated with a temporary liver function disturbance caused by either direct injuries (migrating larvae) or indirect (immunological or eosinophilia) injuries that occur during *Trichinella* infection. Additionally, the apo E expression level strongly depends on the cytokine production pattern. There is a well-known mechanism that proves that some pro-inflammatory cytokines, such as TNF-α, IFN-γ, or IL-1β downregulate apoE production by macrophages [85,86]. Studies, mainly on mice or rats, in which gene expression was measured in various organs and tissues or the native serum cytokine level was assessed, showed that all of these inflammatory mediators were altered during the early and late phases of *Trichinella* infection [82,87,88,89]. Therefore, a shift in cytokine production may also partially affect changes in apoE found in the present study. Finally, it has been shown that oxidative stress significantly modulates adipose tissue and adipocyte apoE expression [90]. Several authors have reported that parasitic infections, including trichinellosis, are accompanied by oxidative stress [91,92,93]. It was demonstrated that, starting from the second or fourth week after infection, blood parameters, such as superoxide dismutase (SOD), glutathione peroxidase (GPx), glutathione S-transferase (GST), and malondialdehyde (MDA), were highly elevated in *Trichinella spiralis*-infected mice and/or rats [93,94,95]. Hence, an oxidative balance disruption should be considered as another explanation of the decreased apoE expression level in serum of experimentally infected pigs in the present trial. 

Serum amyloid P-component precursor was recognized as another protein of interest, whose expression was downregulated and which was successfully identified by mass spectrometry. Its down-expression was found on day 13 postinfection in pigs experimentally infected with *T. pseudospiralis*. Serum amyloid P component (SAP) is synthesized in the liver, then released into the bloodstream, and its half-life is approximately 24 hours [96,97]. SAP, like C-reactive protein (CRP), belongs to the pentraxin family of proteins, which have a pentameric cyclic structure and exhibit calcium-dependent ligand binding [98]. Several, previous studies have shown that SAP binds, among others, to amyloid fibrils and infectious agents, such as pathogenic viruses [99]; different species of bacteria, e.g., *Streptococcus pyogenes*, *Neisseria meningitides,* and *Escherichia coli* [100]; Gram-negative bacterial lipopolysaccharide (LPS) [101] or *Candida albicans* fungal cell surface [102]. It is still unclear whether SAP helps the host or rather protects the pathogen, but it was demonstrated in mice that SAP acts as a positive acute-phase protein that increases almost 50 times during an inflammatory response induced by *Salmonella enteritidis* lipopolysaccharide or croton oil injection [103]. Interestingly, the serum SAP level was also doubled on the second day after the experimental infection of mice with *Nippostrongylus brasiliensis* [104]. On the other hand, only a slightly elevated level of SAP was demonstrated in humans during inflammatory states (i.e., coeliac disease, ulcerative colitis, Crohn’s disease, or rheumatoid arthritis) or neoplastic diseases [105]. Contrary to data mentioned above, in our studies more than 5-fold decrease of amyloid P-component precursor expression level in *T. pseudospiralis*-infected pigs was observed and the cause of this phenomenon remains unknown and enigmatic. Basically, significantly low serum SAP levels have been identified as a characteristic feature in the course of human hepatic diseases [105,106]. Therefore, we link our research results with possible functional disorders of that organ caused by *Trichinella pseudospiralis* invasion. Nonetheless, further studies are necessary to fully understand the role of SAP protein in healthy pigs, as well as in those infected with *Trichinella* spp.

Among six upregulated proteins that we found on day 13 postinfection in pigs experimentally infected with various *Trichinella* species, homeobox protein Mohawk (Mkx, Irxl 1) deserves special attention. In the present study, its overexpression was shown in the serum of *T. britovi*-infected swine. The Mohawk homeobox (*Mkx*) gene represents the three-amino acid loop extension (TALE) superclass of atypical homeobox genes and encodes 353 amino acid protein that has transcriptional repressor activity [107,108]. What we know so far is that Mkx is expressed, mainly during mouse embryogenesis, in progenitor cells of skeletal muscle, tendon, and cartilage, as well as in chords of the male gonad or metanephrogenic kidney [107,109,110]. Several studies have shown that protein Mohawk is crucial for musculoskeletal system development. It acts as a repressor for negative regulatory factors of type I collagen [111] and plays a negative regulatory role in muscle differentiation [108,112]. It is also involved in tendon development [111] and serves as a morphogenic regulator of cell adhesion [107]. In addition, urine proteomic analysis indicated that homeobox protein Mohawk should be considered as a molecule associated with neoplastic process as well [113]. Given that some molecular functions, such as myoblast differentiation suppression via *myoD* expression changes or collagen type I synthesis regulation, are convergent for both excretory-secretory products of *Trichinella* ML and protein Mohawk, we postulate that Mkx can also play a role in muscle tissue remodeling being the host’s endogenous response to infection, however, the exact molecular pathway responsible for the Mkx expression pattern during trichinellosis is impossible to explain in these studies.

### 3.2. Fully Infective Stage of T. spiralis, T. britovi, and T. pseudospiralis Larvae in Pig Striated Muscles (60 Day Postinfection)

2-D electrophoretic analysis of serum samples obtained from pigs on day 60 postinfection revealed that upregulated proteins predominated in the late phase of invasion, particularly when compared to day 13 postinfection. Thus, in the serum of pigs experimentally infected with *T. spiralis*, all of the five altered spots were upregulated on day 60 postinfection, and the molecular weight of the up-expressed proteins ranged from 12.90 kDa to 28.60 kDa. Unfortunately, only one of these spots was successfully identified by the MS technique, namely, lambda free light chain of immunoglobulin. Similar results were observed for *T. britovi*-infected pig sera, in which 5 spots were upregulated while the remaining one was downregulated. Their molecular weight varied between 12.90 and 32.90 kDa, and we were able to identify two of them: lambda light chain of immunoglobulin and apolipoprotein E precursor. On day 60 postinfection, serum samples from pigs infected with *T. pseudospiralis* contained 2 differentially expressed spots, which were identified by the MALDI-TOF MS technique as complement component C3 and apolipoprotein A-I. It is worthwhile to note that other authors have also reported similar difficulties in the identification of protein spots by MS, which may partially be explained by the fact that the quantity of the protein may have been insufficient to generate the correct mass spectrum or the confidence levels of the databases may have been insufficient to obtain unequivocal results [23].

It is particularly worth pointing out that on day 60 postinfection the only proteins whose expression was downregulated in the serum of experimentally infected pigs were those whose functions are strictly related to lipid metabolism. Thus, apolipoprotein E precursor was down-expressed in response to the experimental infection of pigs with *T. britovi*, and possible mechanisms of the infection-induced apoE serum expression changes are widely discussed in the previous section of this manuscript. Similarly, apolipoprotein A-I (Apo A-I) was downregulated in the serum of *T. pseudospiralis*-infected swine on day 60 post-inoculation. The down-expression of Apo A-I may partly be attributed to factors described previously to explain downregulation mechanisms for both apo E and apo J, namely, an elevated level of inflammatory molecules that shut down apo A-I induction, changes in *Trichinella*-induced lipid metabolism and oxidative balance disruption. Besides, in pigs, apo A-I belongs to negative acute-phase proteins [114], thus, its downregulation may also contribute to the progression of the inflammatory process during the muscular phase of the invasion evoked by nonencapsulated genotypes of *Trichinella.* Additionally, several studies have found that HDL and Apo A-I, which is the main component of HDL, show anticoagulant activity [115,116]. Therefore, we postulate that apo A-I down-expression may also be involved in potential blood coagulation and thrombotic disorders during *T. pseudospiralis* infection.

Further, in the late phase of infection (60 d.p.i.), free lambda immunoglobulin light chains were upregulated in the serum of pigs experimentally infected with two different encapsulated species of *Trichinella*, namely *T. spiralis* and *T. britovi*. During the synthesis of immunoglobulins, light chains are produced in excess (40%) to heavy chains and secreted as free light chains (FLC) into the serum, after which they are rapidly cleared and metabolized by the kidneys. Therefore, serum concentrations of FLC depend on the balance between their production and renal clearance as well [117]. Importantly, several FLC biological functions can be indicated, i.e., antiangiogenic, prothrombinase, proteolytic, and complement-activating activities; mast cell activation; or specific binding activity for substances and antigens [118]. It has been shown that an FLC increase in human serum is typically observed in the course of various chronic-inflammatory or autoimmune diseases, such as rheumatoid arthritis [119], rhinitis [120], inflammatory bowel disease [121], atopic dermatitis [122], or certain viral infections [123,124]. Our results suggest that prolonged infection with *Trichinella* and persistent antigen stimulation drive B cell activation, resulting in polyclonal FLC overproduction. Furthermore, an increased lambda FLC expression also clearly indicates that *Trichinella*-induced inflammation has entered a chronic but still immune active phase. The upregulation of lambda FLC found in our studies could, therefore, be considered as a potential biomarker for the late phase of the infection induced by encapsulated species of *Trichinella*.

The last protein identified was complement component C3, whose expression was upregulated on day 60 postinfection, although that upregulation was found only in the serum of *T. pseudospiralis*-infected swine. As is well known, the complement system plays a pivotal role in the innate immune response and three main pathways of its activation, namely classical, alternative, and mannose-binding lectin can be distinguished. Moreover, activation of complement factor C3 is set in the central position in the abovementioned activation routes [125]. Some previous *in vitro* studies have found that the surface of infective larvae of *T. spiralis* binds the C3 component, but not C5 or C9 [126]. Similarly, using an *in vitro* model, Hong et al. [127] have shown that various stages of *Trichinella spiralis*, i.e., adults, newborn, and muscle larvae, are able to activate the complement system, mainly, via the alternative pathway and, to a lesser degree, through the classical one. On the other hand, studies conducted by Stankiewicz et al. [128], based on an *in vivo* mouse model, indicated that C3 binding to *T. spiralis* muscle larvae was severely impaired. In other comprehensive studies, Näreaho et al. [129] observed a strong C3 binding to the stichocytes of *T. spiralis* and *T. nativa* muscle larvae as well as the muscular part of the adult stage, but not to the newborn. Recently, Zhao et al. [130] have shown that calreticulin, a Ca^2+^-binding protein that has been identified on the surface and in secretory products of various *T. spiralis* stages, can bind C1q and, as a consequence, reduce C3 generation. Interestingly, in the present study, no changes in the complement expression level were found in the serum of pigs experimentally infected with *T. spiralis* and *T. britovi* compared to the control group, which may indicate a different inflammatory myopathy pattern between *T. pseudospiralis* and encapsulated species of *Trichinella* triggering the infection. It should be emphasized, however, that from a pathophysiological point of view, molecular, biochemical, and structural changes in the host’s body fluids or tissues caused by particular *Trichinella* species on particular days of infection may not correspond to disorders induced by another *Trichinella* species at the same time points. Different time needed for collagen capsule formation in host striated muscles induced by various encapsulated species of *Trichinella*, or, variation in specific antibody kinetics driven by various *Trichinella* species exposure can serve here as an example. Similarly, using the western-blot technique, Gómez-Moralez et al. [131] observed differential patterns of recognition of crude worm extracts from various species of *Trichinella* muscle larvae by specific swine IgG antibodies. Moreover, studies conducted by Bruschi et al. [132] have also demonstrated differences in the host inflammatory response against encapsulated and nonencapsulated *Trichinella* species. On the other hand, in our studies, different types of protein, often unique for particular groups of pigs experimentally infected with various *Trichinella* species, may also have resulted from different doses used for experimental infection and different muscle larvae burden, and hence, different experiment conditions.

## 4. Materials and Methods 

### 4.1. Ethics Statement

Permission for the present studies was granted by the 2nd Local Ethics Committee for Animal Experimentation at the University of Life Sciences in Lublin-Resolution No. 73/2017.

### 4.2. Parasites

Three different *Trichinella* species (genotypes), namely *Trichinella spiralis* (T1), *Trichinella britovi,* (T3) and *Trichinella pseudospiralis* (T4), were used in these studies. *Trichinella spiralis* ML and *Trichinella britovi* ML were isolated in Poland during routine meat inspections from a naturally infected pig and a wild boar, respectively. Subsequently, both of these isolates were identified at the species level by multiplex chain reaction (multiplex PCR) according to Zarlenga et al. [133]. *Trichinella pseudospiralis* was a kind gift from the Witold Stefański Institute of Parasitology, Polish Academy of Sciences. Before the experiment, all of the abovementioned strains had been passaged once through a domestic pig (Pulawska x Polish Large White breed; one pig/per strain).

### 4.3. Pig Infection

The studies involved 24 healthy young Pulawska/Polish Large White crossbreed pigs (both sexes, aged 10 weeks, average body weight 20 kg) purchased from a farm without any known *Trichinella* infection history. A serological test (Priocheck Trichinella Ab) was also performed to confirm the *Trichinella*-negative status of these pigs. Before the experimental inoculation, swine fecal samples were examined for parasite eggs by the flotation method with saturated sodium chloride solution and decantation.

*T. spiralis*, *T. britovi,* and *T. pseudospiralis* muscle larvae were recovered by digestion from the muscles of the swine on which the parasites had been passaged (see paragraph before). Larvae displaying motility were counted (each species separately) and suspended in 30% gelatin blocks. Pigs were randomly divided into three experimental groups (T1, T3, and T4) of 6 pigs each, and one control group (C) of 6 pigs as well. Pigs were infected by administering a single dose *per os* of: 1000 *T. spiralis* ML/per pig (group T1); 2000 *T. pseudospiralis* ML/per pig (group T4); and 3000 *T. britovi* ML/per pig (group T3). The infective dose (the number of *Trichinella* muscle larvae used for experimental infection) was based on global literature data describing reproductive capacity index (RCI) of various *Trichinella* species [14] as well as on our previous pilot studies results. The control group was given the pure gelatin capsules without any *Trichinella* larvae. During the experiment, pigs were housed in four separate units with free access to water.

### 4.4. Blood Recovery

Two different terms corresponding to the intestinal and muscular phase of the infection were selected for blood collection from the infected and control pigs. Blood samples from each pig in each group were collected from the right external jugular vein at 13 and 60 days postinfection (d.p.i.). To obtain serum, the blood samples were centrifuged at 2000× *g* for 10 min at 4 °C, and, then serum samples were placed into Eppendorf tubes (1.5 mL) and frozen at −80 °C until further analysis. 

### 4.5. Larval Recovery and Counting

Pigs were sacrificed 62 days after experimental inoculation. Their average body weight after slaughter was 37.5 kg.

The muscle larvae intensity was determined by the digestion procedure according to European Commission Regulation EU 2015/1375 [16]. Samples intended for the digestion procedure were collected from the diaphragm and the tongue. Muscles of total weight under 50 g were digested entirely, whereas those over 50 g were cropped to form 50 g specimens and then digested. The number of muscle larvae per gram of muscle tissue (lpg) was presented as an average calculated separately for each group and each muscle examined.

In addition, muscle larvae isolated from the diaphragm of each pig were identified at the species level by multiplex PCR, and no mixed infections or contaminations were found in any of the experimental groups.

### 4.6. Swine Serum Sample Preparation for 2-Dimensional Gel Electrophoresis (2-DE)

To reduce the concentration of high-abundance proteins and increase the concentration of low- and medium-abundance ones, serum samples were processed by the ProteoMiner Protein Enrichment Large-Capacity Kit (Bio-Rad, Hercules, CA, USA) in accordance with the manufacturer’s instruction. Depleted samples were then precipitated with 4 volumes of ice cold acetone at −20 °C for 2 h and centrifuged for 60 min at 20,000× *g* at 4 °C. Protein pellets obtained were dissolved in a lysis buffer containing 7 M urea, 2 M thiourea, 4% w/v 3-[(3-cholamidopropyl) dimethylammonio]-1-propanesulfonate (CHAPS), 1% w/v dithiothreitol (DTT), 0.2% w/v 3–10 carrier ampholytes, and 2 mM tributylphosphine. The protein concentration was determined using a modified Bradford assay (Bio-Rad) according to the manufacturer’s instructions.

### 4.7. 2-DE

Serum protein samples (500 µg) were applied to 3–10, 11 cm nonlinear ReadyStrip IPG Strips (Bio-Rad). Isoelectrofocusing (IEF) was performed using a Protean i12 IEF Cell (Bio-Rad) as follows: (i) 250 V for 125 Vh, (ii) 500 V for 250 Vh, (iii) 1000 V for 500 Vh, (iv) linear increase to 3500 V for 1.5 h, and (v) 3500 V for 40,000 Vh. Subsequently, the IPG strips were reduced for 15 min with 1% w/v DTT in the equilibration buffer (6 M urea, 0.5 M Tris/HCl, pH 6.8, 2% w/v SDS, 30% w/v glycerol) and then alkylated for 20 min in an equilibration buffer with 2.5% w/v of iodoacetamide. The second dimension of electrophoresis was performed at 40 V for 2.5 h and then at 90 V for 15 h at 15 °C with 12% polyacrylamide gels in a Tris-glycine buffer (25 mM Tris/HCl, 192 mM glycine, 0.1% SDS) using a Protean Plus Dodeca Cell electrophoresis chamber (Bio-Rad). After 2-DE separation, the gels were stained with colloidal Coomassie Brilliant Blue G-250 (Sigma-Aldrich) as previously described by Pink et al. [134].

### 4.8. Image Analysis

Gel image acquisition was carried out using a GS-800 Calibrated Densitometer (Bio-Rad). 2-D gel images were analyzed using PDQuest Advanced 2D-Gel Analysis 8.0.1. Software (Bio-Rad). Image filtration, detection of spots, intensity quantification, background subtraction, and spot matching between gels were performed automatically. Next, manual verification was performed to remove any false spots and add the missed one. The spots present on at least four gels from the experimental or control groups were designated as expressed protein spots and further analyzed. The gel with the highest number of spots was used as the master gel for matching the remaining ones. The spot volume (expressed as spot optical density-OD) was used as a parameter for quantifying protein expression after normalization based on the local regression model (LOESS). After normalization, the volume of each spot was calculated as the average from two replicates of each biological sample. Statistically significant spots (*P* ˂ 0.05, statistical analysis-see the paragraph below) were considered for further analysis. The degree of difference between protein groups was expressed as an average ratio. To measure variability within the groups, the coefficients of variation (CV) were calculated. The experimental isoelectric point (pI) and molecular weight (kDa) values were computed on the basis of standard 2-D markers for each protein spot identified.

### 4.9. Mass Spectrometry and Bioinformatics Data Analysis

#### 4.9.1. In-Gel Digestion of Proteins

Protein spots that showed significantly altered expression were excised from the gels, decolorized by washing in a buffer containing 25 mM NH_4_HCO_3_ in 5% v/v acetonitrile (ACN) and then washed two times in a solution of 25 mM NH_4_HCO_3_ in 50% v/v ACN. The gel pieces were dehydrated in 100% ACN, vacuum dried and digested overnight at 37 ℃ in a trypsin solution (8 µL/spot of 12.5 µg trypsin/mL in 25 mM NH_4_HCO_3_; Promega, Madison, WI, USA) at 37 °C.

#### 4.9.2. Matrix-Assisted Laser Desorption/Ionization Time-of-Flight Mass Spectrometry (MALDI TOF MS) Analysis

Mass spectra were acquired in the positive-ion reflector mode using a Microflex MALDI-TOF (matrix-assisted laser desorption/ionization time-of-flight) mass spectrometer (Bruker Daltonics, Germany).

The resulting peptides were extracted with 100% ACN, combined with an equal volume of matrix solution (5 mg/mL of α-cyano-4-hydroxycinnamic acid (CHCA), 0.1% v/v of trifluoroacetic acid (TFA), 50% v/v ACN) and then loaded onto a MALDI-MSP AnchorChip 600/96 plate (Bruker Daltonics, Germany) in a final volume of 1 µL. Droplets were allowed to dry at room temperature. Peptide Mass Standard II (Bruker Daltonics) with a mass range of 700–3200 Da was used for mass scale calibration. Mass spectra were acquired with 150 shots of a nitrogen laser operating at 20 Hz and were internally calibrated using porcine tryptic autolytic products (842.51 and 2211.10 *m/z*).

Peptide mass fingerprinting (PMF) data were compared with the Uniprot or NCBI mammalian protein databases using the MASCOT search engine. The following parameters were applied for database searching: trypsin as an enzyme with one missed cleavage allowed, carbamidomethylation of cysteine as a fixed modification, methionine oxidation as a variable modification, and 150 ppm mass accuracy. The results of PMF-based analysis (identification) were considered acceptable if the protein score was significant (*P* ˂ 0.05) with at least four matching peptides and 10% peptide coverage.

### 4.10. Statistical Analysis

Student’s *t*-test was used to compare the intensity of *Trichinella spiralis*, *Trichinella britovi,* and *Trichinella pseudospiralis* infection between the diaphragm and the tongue in each infected group, and these calculations were performed with the Statistica 9.1 software. Previously, Shapiro–Wilk’s test of normality and Levene’s test of equal variances had been performed.

Student’s *t*-test (two-tailed), integrated with the PDQest software, was also performed to calculate differences in serum protein expression between the groups (C vs. T1, C vs. T3, and C vs. T4) for both sampling time points (i.e., 13 and 60 d.p.i.).

For all these analyses, the level of significance was set at *P* ˂ 0.05.

## 5. Conclusions

This is the first report on quantitative protein profile assessment of serum obtained from pigs experimentally infected with various *Trichinella* species during two different phases of the invasion. The results of the present study prove that infection with *T. spiralis*, *T. britovi,* and *T. pseudospiralis* is able to induce changes in the serum proteomic profile of experimentally infected pigs. It seems that pattern of these changes depends on the *Trichinella* species triggering the infection and the infection phase as well. Further, in the case of pigs experimentally infected with encapsulated species of *Trichinella*, a significantly higher magnitude of changes in protein expression was observed on day 60 postinfection (mean fold changes of 10.04 and 10.45 for *T. spiralis* and *T. britovi*, respectively) compared to day 13 postinfection (mean fold changes of 2.84 and 1.33 for *T. spiralis* and *T. britovi*, respectively). This phenomenon indicates disease progression and suggests that the parenteral phase of the invasion, characterized by fully mature and encapsulated larvae settled in the host striated muscles, induces much stronger alterations in the swine serum proteome. Our study also shows that infection with *Trichinella spiralis*, *Trichinella britovi,* and *Trichinnella pseudospiralis* causes lipid metabolism disorders manifested by downregulation of apo A-I or apo E or apo J expression in the serum of infected pigs in either the early or the late phase of the invasion. Moreover, depending on the *Trichinella* species triggering the infection, proteins involved in the immune response, inflammatory response, blood coagulation process, collagen production, and muscle differentiation were also differentially expressed. Finally, several protein spots, in particular those whose expression was altered on day 60 postinfection, could not be identified by MALDI-TOF MS, and, therefore, further studies using more sophisticated proteomic techniques are needed to better understand host–parasite interactions and identify new biomarkers for early diagnosis of *Trichinella*-infection in pigs.

## Figures and Tables

**Table 1:** Distribution and Intensity of *Trichinella* Larvae Infection in Diaphragms and Tongues of Pigs Experimentally Infected with *T. spiralis*, *T. britovi,* and *T. pseudospiralis*.

Experimental Group	Numbers of *Trichinella* Larvae/g Muscle (lpg)						
	Diaphragm (Pillars)		Tongue		*P*-Value	Diaphragm Pillars and Tongue (Together)	
	Mean	SD	Mean	SD		Mean	Min–Max
*T. spiralis* (n = 6)	89.52 ^a^	60.90	93.07 ^a^	40.63	0.9078	91.29	42.67–177.30
*T. britovi* (n = 6)	41.46 ^a^	20.28	37.75 ^a^	19.58	0.7536	39.60	22.26–77.01
*T. pseudospiralis* (n = 6)	34.20 ^a^	32.43	19.77 ^a^	21.68	0.3861	26.98	0.22–65.27
Control (n = 6)	0.00	0.00	0.00	0.00	-	0.00	-

Student’s *t*-test was used to compare mean lpg for the tongue and the diaphragm within particular experimental groups of pigs. “a”—means within a row followed by the same letter are not significantly different. SD: standard deviation.

**Table 2:** Summary of differentially expressed protein spots identified by matrix-assisted laser desorption/ionization time-of-flight mass spectrometry (MALDI-TOF MS) analysis in the blood serum of pigs infected with *Trichinella spiralis* (T1), *Trichinella britovi* (T3), and *Trichinella pseudospiralis* (T4) compared with the control group (C) 13 days after infection. Spot numbers correspond to those in Figure 1; Figure 2.

Spot No.	Accession No. UniProt/NCBI	Protein Name	CC	Fold Change	SC ^1^ (%)/ MS ^2^	PM ^3^	Theo. pI/Mw ^4^	Exp. pI/Mw ^5^
*T. spiralis-*infected group (T1) vs. Control								
4801/1	BAM66301	IgM heavy-chain constant region, partial	E	−2.55	60/195	15	5.74/49.94	5.20/83.90
3402/2	NP_001123430	Antithrombin-III precursor	E	−1.52	49/184	20	5.84/52.87	4.40/56.10
9601/3	AAA51295	Immunoglobulin gamma-chain	E	3.28	25/93	7	6.71/51.89	8.90/50.90
1602/4	-	Unidentified protein	-	5.29	-	-	-	4.10/39.90
2201/5	Q29549	Clusterin	E	−1.56	13/73	5	5.62/52.31	4.20/35.00
*T. britovi-*infected group (T3) vs. Control								
3801/1	BAM66301	IgM heavy-chain constant region, partial	E	1.39	58/200	13	5.74/49.94	4.80/84.00
9008/2	-	Unidentified protein	-	1.08	-	-	-	7.60/23.90
9009/3	XP_001507016	Homeobox protein Mohawk, partial	N	1.51	20/80	8	9.88/37.06	8.40/23.90
*T. pseudospiralis*-infected group (T4) vs. Control								
1602/1	-	Unidentified protein	-	3.25	-	-	-	4.10/39.90
0302/2	Q29549	Clusterin	E	−2.31	21/67	7	5.62/52.31	3.80/40.10
1202/3	-	Unidentified protein	-	−2.59	-	-	-	4.10/35.70
2201/4	Q29549	Clusterin	E	−1.52	13/73	5	5.62/52.31	4.20/35.00
6101/5	NP_999473	Apolipoprotein E precursor	CP	−4.28	39/117	12	5.62/36.63	5.40/33.00
9116/6	NP_999052	Serum amyloid P-component precursor	E	−5.68	38/110	8	8.74/25.74	9.30/25.50

SC = sequence coverage; MS = MASCOT score; PM = peptides matched; CC = cellular component, E = extracellular, CP = cytoplasm. ^1^ The percentage of sequence coverage. ^2^ The MASCOT score. ^3^ The number of peptides matched. ^4^ Theoretical molecular weight (Mw) and isoelectric point (pI) values based on the Uniprot/NCBI databases. ^5^ Experimental molecular weight (Mw) and isoelectric point (pI) values computed for each identified protein spot based on the standard 2-D markers.

**Table 3:** Summary of differentially expressed protein spots identified by MALDI-TOF MS analysis in the blood serum of pigs infected with *Trichinella spiralis* (T1), *Trichinella britovi* (T3), and *Trichinella pseudospiralis* (T4) compared with the control group (C) 60 days after infection. Spot numbers correspond to those in Figure 3; Figure 4.

Spot No.	Accession No. UniProt/NCBI	Protein Name	CC	Fold Change	SC ^1^ (%)/ MS ^2^	PM ^3^	Theo. pI/Mw ^4^	Exp. pI/Mw ^5^
*T. spiralis-*infected group (T1) vs. Control								
1101/1	P01846	Ig lambda chain C region OS	E	2.48	68/73	4	6.75/11.17	4.60/28.60
9007/2	-	Unidentified protein	-	14.32	-	-	-	9.60/26.60
9006/3	-	Unidentified protein	-	8.08	-	-	-	9.40/24.80
9001/4	-	Unidentified protein	-	14.87	-	-	-	9.50/13.60
9002/5	-	Unidentified protein	-	10.47	-	-	-	9.50/12.90
*T. britovi-*infected group (T3) vs. Control								
5101/1	NP_999473	Apolipoprotein E precursor	CP	−1.64	44/194	15	5.62/36.63	6.30/32.90
1101/2	P01846	Ig lambda chain C region OS	E	2.21	68/73	4	6.75/11.17	4.60/28.60
3004/3	-	Unidentified protein	-	3.29	-	-	-	5.00/26.00
3005/4	-	Unidentified protein	-	10.40	-	-	-	4.80/21.70
9007/5	-	Unidentified protein	-	17.13	-	-	-	9.60/26.60
9002/6	-	Unidentified protein	-	28.03	-	-	-	9.50/12.90
*T. pseudospiralis-*infected group (T4) vs. Control								
4301/1	XP_020936478	Complement C3 isoform X1	E	1.53	11/83	13	5.99/19.30	5.20/43.30
3001/2	AAA30992	Apolipoprotein A-I	E	−1.87	51/219	18	5.38/30.31	4.90/23.50

SC = sequence coverage; MS = MASCOT score; PM = peptides matched; CC = cellular component, E = extracellular, CP = cytoplasm. ^1^ The percentage of sequence coverage. ^2^ The MASCOT score. ^3^ The number of peptides matched. ^4^ Theoretical molecular weight (Mw) and isoelectric point (pI) values based on the Uniprot/NCBI databases. ^5^ Experimental molecular weight (Mw) and isoelectric point (pI) values computed for each identified protein spot based on the standard 2-D markers.
